# Impact of MnTBAP and Baricitinib Treatment on Hutchinson–Gilford Progeria Fibroblasts

**DOI:** 10.3390/ph15080945

**Published:** 2022-07-29

**Authors:** Elena Vehns, Rouven Arnold, Karima Djabali

**Affiliations:** Epigenetics of Aging, Department of Dermatology and Allergy, TUM School of Medicine, Munich Institute of Biomedical Engineering, Technical University of Munich (TUM), 85748 Garching, Germany; elena.vehns@tum.de (E.V.); rouven.arnold@tum.de (R.A.)

**Keywords:** progerin, MnTBAP, baricitinib, JAK-STAT, senescence, lamin

## Abstract

Hutchinson–Gilford progeria syndrome (HGPS) is a rare premature aging disease. It is caused by a mutation in the *LMNA* gene, which results in a 50-amino-acid truncation of prelamin A. The resultant truncated prelamin A (progerin) lacks the cleavage site for the zinc-metallopeptidase ZMPSTE24. Progerin is permanently farnesylated, carboxymethylated, and strongly anchored to the nuclear envelope. This leads to abnormalities, such as altered nuclear shape, mitochondrial dysfunction, and inflammation. HGPS patients display symptoms of physiological aging, including atherosclerosis, alopecia, lipodystrophy, and arthritis. Currently, no cure for HGPS exists. Here we focus on a drug combination consisting of the superoxide dismutase mimetic MnTBAP and JAK1/2 inhibitor baricitinib (Bar) to restore phenotypic alterations in HGPS fibroblasts. Treating HGPS fibroblasts with the MnTBAP/Bar combination improved mitochondrial functions and sustained Bar’s positive effects on reducing progerin and pro-inflammatory factor levels. Collectively, MnTBAP/Bar combination treatment ameliorates the aberrant phenotype of HGPS fibroblasts and is a potential treatment strategy for patients with HGPS.

## 1. Introduction

Hutchinson–Gilford progeria syndrome (HGPS, OMIM 176670) is a premature aging disease that belongs to the family of laminopathies [1]. It affects approximately 1 in 20 million people [2]. This syndrome is caused by a mutation in the *LMNA* gene, encoding nuclear lamin A and C isoforms [3,4]. In most cases, a de novo single-base substitution G608G (c.1824; GGC>GGT; p.G608G) occurs, resulting in a cryptic splice site within exon 11. This aberrant splicing leads to a 50-amino-acid truncation of prelamin A, and the resulting shortened protein is called progerin [3,5]. Progerin lacks the cleavage site for the zinc-metallopeptidase ZMPSTE24. Consequently, progerin is permanently farnesylated, carboxymethylated, and strongly anchored to the nuclear envelope [6,7,8]. This leads to abnormalities in HGPS nuclei, such as altered nuclear shape and blebbing [3,9]. HGPS cells also show other defects, such as decreased cell proliferation, abnormal gene expression, accumulation of reactive oxygen species (ROS), mitochondrial dysfunction, and premature senescence entry [10,11,12]. HGPS patients display many symptoms of physiological aging, including atherosclerosis, alopecia, lipodystrophy, joint contractures, bone abnormalities, and severe cardiovascular disease, leading to stroke and heart attack [1,13]. The latter is the most common cause of death of patients with HGPS at an average age of 14.6 years [8].

Currently, no cure for HGPS exists, although several therapeutic strategies have been developed to improve the clinical features and lifespan of patients with HGPS [14]. The first strategy aims to block the farnesylation of prelamin A using a farnesyltransferase inhibitor (FTI, lonafarnib) [15]. Lonafarnib prevents nuclear blebbing and reduces progerin anchorage at the nuclear membrane, resulting in the restoration of normal nuclear morphology [15,16,17]. In 2007, the FTI lonafarnib was tested in the first clinical trial involving HGPS patients [18,19]. The treatment increased the mean individual survival by 1.6 years, increased body weight, decreased skeletal rigidity, and improved bone mineral density in patients with HGPS [8,19]. Nevertheless, lonafarnib also leads to side effects such as diarrhea, nausea, and anorexia [20]. Lonafarnib was approved by the US Food and Drug Administration (FDA) in 2020 [21]. While FTI improves some features of HGPS symptoms, it is not a cure. Consequently, further attempts to restore HGPS cellular defects are required.

Another promising therapeutic strategy for alleviating HGPS symptoms is related to the observation that HGPS patients develop various pathologies that also occur during normal aging. Several genes associated with four pathologies, namely, atherosclerosis, arthritis, alopecia, and lipodystrophy, have been identified and are linked to the Janus kinase (JAK), a signal transducer and activator of a transcription (STAT) signaling pathway [12].

The JAK/STAT pathway controls cell proliferation, apoptosis, and inflammation [22]. Its dysregulation can cause chronic inflammation, as observed in age-related diseases such as arthritis or atherosclerosis [12,22,23]. In HGPS fibroblasts, JAK/STAT signaling is over-activated during replicative senescence [12]. The FDA has approved JAK1/2 inhibitor baricitinib (Bar) for the treatment of rheumatoid arthritis [24]. Bar delays senescence, significantly decreases proinflammatory factor levels, and restores cell homeostasis in HGPS cells [12]. Furthermore, progeroid *Zmpste24*^–/–^ mice treated with another JAK1/2 inhibitor, ruxolitinib, show reduced bone fracturing, grip strength, and bone mineral content, and increased survival [25].

In addition to inflammation, studies point to mitochondrial dysfunctions in HGPS cells [12,26,27,28]. Energy production is the main function of the mitochondrion, but the organelle is also involved in cellular homeostasis and metabolism [29]. It is not surprising that mitochondria play a central role in the aging process, considering their dominant role in metabolism [30]. Mitochondrial dysfunction is characterized by dysregulation of ATP production, the release of pro-apoptotic products, and increased ROS formation [29]. These changes are also observed during physiological aging [31]. High ROS levels can lead to oxidative stress, resulting in organ injury [32,33]. Superoxide anions (O_2_^−^), one of the ROS species, play an important role in inflammation [34,35]. They are responsible for the regulation of cytokines, recruitment of neutrophils, and production of chemotactic factors [36,37]. Excessive O_2_^−^ levels, associated with acute or chronic inflammation, may lead to O_2_^−^ -mediated-damage [32]. Superoxide dismutase (SOD) neutralizes O_2_^−^ by reducing it to hydrogen peroxide (H_2_O_2_) [38]. There are a few known antioxidant compounds that ameliorate the HGPS phenotype and reduce oxidative stress and progerin level [39]. The treatment of HGPS cells with methylene blue (MB) or magnesium reduces the ROS level or ATP availability [40,41,42]. In addition, the ROS-scavenger *N*-acetyl cysteine (NAC) or the antioxidant sulforaphane (SFN) ameliorates the growth rate, the nuclear shape, and autophagy level in HGPS fibroblasts [39,43,44,45]. However, these compounds were used at high concentrations and show cytotoxicity, thus limiting their use in combination treatment [41,42,44,45]. Mn (III) tetrakis (4-benzoic acid) porphyrin (MnTBAP) is a SOD mimetic that protects against oxidative stress by scavenging O_2_^−^, H_2_O_2_, peroxynitrite ions (ONOO), and lipid-peroxyl free radicals [32,46,47]. It also prevents the development of DNA single-strand breaks and acts as an antioxidant [48,49]. In this study, we investigated the effect of MnTBAP on HGPS fibroblasts because of its broader effect on ROS, mitochondrial function, and intracellular stress levels [48,49]. Since HGPS cells exhibit mitochondrial dysfunction, we evaluated whether MnTBAP could restore this function in HGPS fibroblasts. 

The synergistic effects of two drugs with different modes of action can be more efficacious than a single drug, permitting the use of much lower doses, and thus possibly reducing the risks of side effects [50]. Indeed, high concentrations of JAK inhibitors can reduce neutrophil numbers and block immune responses [51,52]. In the current study, we evaluated the effect of MnTBAP, alone and in combination with Bar, on HGPS fibroblasts. We examined whether MnTBAP could restore mitochondrial function in HGPS cells, and whether Bar could act synergistically to ameliorate cellular homeostasis and morphology in HGPS cells. We show that MnTBAP/Bar partially restores mitochondrial functions in HGPS fibroblasts, making MnTBAP/Bar a potential combination treatment for HGPS. 

## 2. Results

### 2.1. Combined MnTBAP/Bar Treatment Ameliorates HGPS Fibroblast Growth Rate and Decreases Replicative Senescence to a Range Similar to Each Drug Alone

MnTBAP and Bar are two drugs that act on different signaling pathways in the cells. MnTBAP is a cell-permeable SOD mimetic and peroxynitrite scavenger [47], while Bar is a small molecule that specifically and reversibly inhibits JAK1 and JAK2 [24,53]. Consequently, Bar blocks the activation of STAT family transcription factors that control the expression of genes implicated in inflammation, cell proliferation, and cell growth [53,54]. First, we optimized the concentration of MnTBAP to be used to avoid drug cytotoxicity in the control and HGPS fibroblasts (Figure S1).

**Figure 1:**
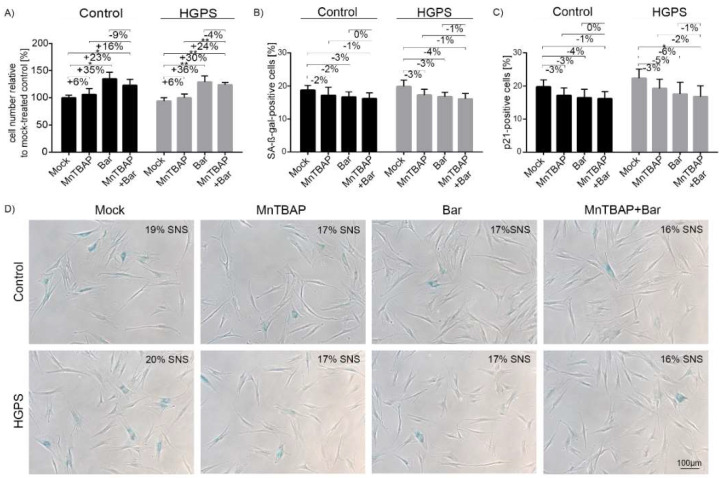
Analysis of the control and HGPS fibroblasts after MnTBAP/Bar treatment. (**A**) The numbers of control and HGPS cells relative to mock-treated control cells. Starting cultures with the same senescence index (approximately 15% SNS) were treated for 9 d with mock, 5 µM MnTBAP, 1 µM Bar or the MnTBAP/Bar combination. (**B**) Percentage of SA-β-Gal-positive cells after treatment. (**C**) Percentages of p21 positive cells after treatment. (**D**) Representative images of SA-β-Gal-stained cells after treatments. At least 900 nuclei were counted for each condition. The data are shown as the mean ± SD (*n* = 4; * *p* < 0.05, ** *p* < 0.01).

The control and HGPS cells were treated with mock or increasing concentrations of MnTBAP (2.5–50 µM) for 4 d, with the medium changed every other day. On day 4, cell counts were determined (Figure S1). The experiments indicated that 5 µM MnTBAP was the optimal concentration, as it maintained the growth rate of the control and HGPS cells and caused no toxicity relative to the mock-treated counterparts (Figure S1). This concentration is also consistent with a recently published concentration [55]. Consequently, 5 µM MnTBAP was used in further experiments. Bar was used at 1 µM following a previous report demonstrating that 1 µM Bar ameliorates several alterations of the phenotypic traits of HGPS cells [12]. Next, we determined the effect of combined MnTBAP and Bar (MnTBAP/Bar) treatment on the growth of the control and HGPS cells (Figure 1A).

The control and HGPS cultures (15% SNS) were treated with single drugs or their combination for 9 d (Figure 1A). The MnTBAP treatment slightly enhanced the proliferation of both the control and HGPS cells (Figure 1A). In addition, as previously reported, Bar alone increased the growth rate of both cell types [12], with a similar effect observed with a combination of MnTBAP/Bar (Figure 1A). The percentage of senescence was determined in parallel with the growth rate (Figure 1B,D). All treatments slightly decreased the number of SA-β-Gal positive cells on day 9, but the change was not significant (Figure 1B,D). Since SA-β-Gal is a late senescence marker, we also determined the levels of the early senescence marker p21. We treated the control and HGPS cells as above for 9 d and scored the p21-positive cells’ by immunocytochemistry (Figure 1C and Figure S2). The MnTBAP/Bar treatment reduced the percentage of p21-positive HGPS cells. This indicated a delayed senescence entry, an effect that could not be observed using the late senescence marker SA-β-Gal. 

### 2.2. Treatment with Bar Inhibits STAT1/2 Signaling and Reduces the Expression of Pro-Inflammatory Cytokines

**Figure 2:**
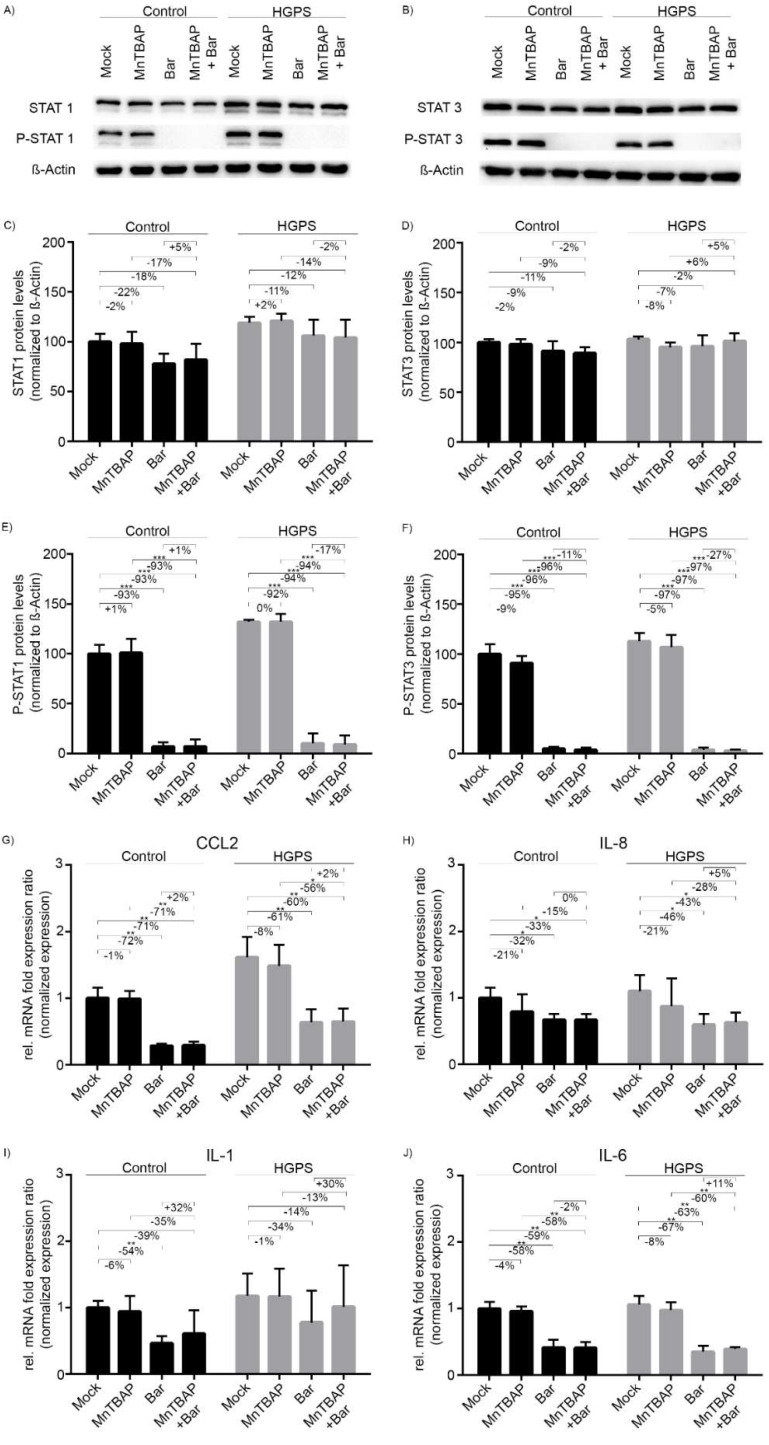
MnTBAP/Bar treatment reduces inflammation. (**A**,**B**) Representative Western blots detecting STAT1, P-STAT1, STAT3, and P-STAT3, normalized to β-actin. The control and HGPS fibroblasts were treated for 9 d with a mock solution, 5 µM MnTBAP, 1 µM Bar, or the MnTBAP/Bar combination. (**C**) Quantification of STAT1, (**D**) STAT3, (**E**) P-STAT1, and (**F**) P-STAT3 signals. Relative mRNA levels of CCL2 (**G**), IL-8 (**H**), IL-1 (**I**), and IL-6 (**J**). Relative expression was normalized to that of GAPDH. Graphs show the mean ± SD (*n* = 3; * *p* < 0.05, ** *p* < 0.01, *** *p* < 0.001).

Altered cellular communication associated with the development of chronic inflammation is one of the hallmarks of aging [56,57]. Recently, we have demonstrated that altered expression of genes encoding pro-inflammatory factors in HGPS cells correlates with an overactivation of the JAK/STAT pathway [12]. Accordingly, we tested whether MnTBAP and Bar treatments could normalize JAK/STAT signaling in HGPS cells. We treated the control and HGPS cultures that had reached 15% SNS for 9 d with MnTBAP and Bar alone or in combination and evaluated the STAT1/3 status of the cells (Figure 2A–F). 

MnTBAP did not affect the total STAT1/3 levels (Figure 2A–F). By contrast, the total STAT1/3 levels tended to decrease upon treatment with Bar and the drug combination, although the change was not significant (Figure 2A–F). On the other hand, Bar and MnTBAP/Bar reduced P-STAT1 levels significantly by approximately 93% and P-STAT3 levels by 96% in both control and HGPS cells (Figure 2A,B,E,F). MnTBAP alone had no noticeble effect on the status of these transcription factors. These data indicated that Bar prevents the phosphorylation and activation of STAT1 and STAT3, and that MnTBAP does not affect these factors.

The JAK/STAT signaling pathway is involved in various cellular mechanisms, such as proliferation, apoptosis, and inflammation [22], and the JAK1/2-inhibitor Bar reduces the expression of proinflammatory genes in HGPS cells [12]. Since we had shown that Bar reduces P-STAT1/3 levels and MnTBAP had no effect, we next evaluated the gene expression profile of several cytokines that are upregulated during replicative senescence [12]. We treated HGPS and control fibroblasts (~15% SNS) for 9 d with MnTBAP, Bar, or a combination of both. The mRNA levels of CCL2, IL-6, and IL-8 were reduced by Bar and MnTBAP/Bar treatments (Figure 2G–J). The mRNA levels of CCL2 were reduced to a similar level by Bar (Ctr: −72%; HGPS −61%) and MnTBAP/Bar treatments (Ctr: −71%; HGPS: −60%) (Figure 2G). Similarly, the levels of IL-6 mRNA were reduced by Bar (Ctr: −58%; HGPS: −67%) and MnTBAP/Bar treatments (Ctr: −59%; HGPS: −63%) (Figure 2J). For IL-8 the reduction was less pronounced and the reduction in IL-1α was not statistically significant (Figure 2H,I). Hence, MnTBAP/Bar significantly reduced CCL2, IL-6, and IL-8 mRNA levels in both cell types, and these effects appeared driven by Bar.

### 2.3. Combined MnTBAP/Bar Treatment Improves Progerin Clearance and Nuclear Morphology to a Range Similar to Bar Alone

**Figure 3:**
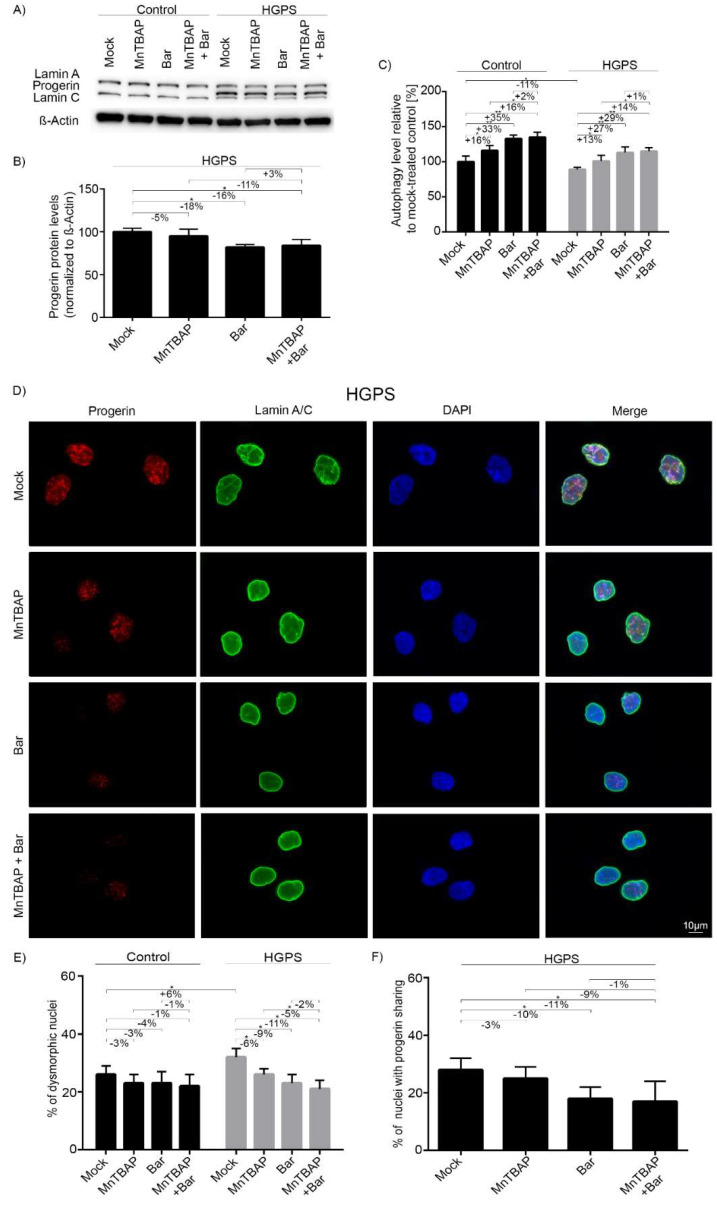
Combined MnTBAP/Bar treatment reduces nuclear abnormalities. (**A**) Representative images of Western blots of lamin A/C after MnTBAP/Bar treatment. Control and HGPS fibroblasts were treated for 9 d with a mock solution, 5 µM MnTBAP, 1 µM Bar, or the MnTBAP/Bar combination. (**B**) Quantification of progerin levels in HGPS cells. (**C**) Autophagy activity was determined by MDC levels using fluorescence photometry. (**D**) Representative immunofluorescence images of HGPS fibroblasts after 9 d of treatment. The cells were stained with anti-progerin (red) and anti-lamin A/C (green) antibodies, and counterstained with DAPI. The number of dysmorphic nuclei (**E**) and cells with high progerin levels (**F**) are indicated. At least 900 nuclei were counted for each condition. Graphs show the mean ± SD (*n* = 4; * *p* < 0.05, ** *p* < 0.01).

The accumulation of progerin in the HGPS nuclear compartment prematurely drives cellular senescence [12,26,58]. Therefore, we assessed the progerin levels in cells after 9 d of treatment with the different regimens (Figure 3A,B). 

As expected, progerin was not detected in control fibroblasts (Figure 3A). In HGPS cells, the MnTBAP treatment reduced progerin by 5%, Bar by 18%, and MnTBAP/Bar by 16% (Figure 3A,B). Collectively, these findings indicate that progerin clearance in the combined drug treatment is mostly attributable to Bar. Autophagy is involved in the increased clearance of progerin [39,59]. Therefore, we monitored autophagy levels in treated cells (Figure 3C). MnTBAP increased autophagy by an average of 16% in control and 12% in HGPS cells (Figure 3C). In agreement with a previous study [12], Bar treatment increased autophagy by 33% in control cells and by 24% in HGPS cells [12]. The drug combination resulted in the highest increase in autophagy (Ctr: 35%; HGPS 26%) (Figure 3C), indicating a positive effect of the two drugs on autophagic activity. Collectively, these data demonstrated that progerin degradation occurs concomitantly with increased autophagy levels, as reported previously [12].

Accumulation of farnesylated progerin is toxic to the cell, and it leads to dramatic changes in nuclear architecture, including nuclear blebbing and micronuclei formation in HGPS cells [9]. These nuclear abnormalities were also observed in control cells, albeit at a lower frequency (Figure 3E and Figure S3). According to a previous study, Bar treatment can ameliorate the defects of HGPS nuclear morphology [12]. We investigated whether MnTBAP could rescue the nuclear envelope alterations in HGPS cells. Treated cells were analyzed by immunocytochemistry with anti-progerin and anti-lamin A/C antibodies (Figure 3D and Figure S3). Progerin was not detected in control cells (Figure S3). In HGPS cells, the accumulation of progerin in the nucleus was clearly associated with the presence of dysmorphic nuclei (Figure 3D). In HGPS cultures, the frequency of occurrence of dysmorphic nuclei was decreased by 6% by MnTBAP, 9% by Bar, and 11% by the treatment combination (Figure 3D,E). In control cells, all tested regimens also reduced the incidence of altered nuclear shape, but the effect was not as pronounced as in HGPS cells (Figure 3E and Figure S3). We also scored the number of brightly labeled progerin-positive nuclei in HGPS cultures (Figure 3D,F). Their numbers were reduced by all treatment regimens (MnTBAP: −3%; Bar: −10%; MnTBAP/Bar: −11%). Taken together, MnTBAP/Bar combination treatment induced a reduction in the nuclear dysmorphism in HGPS cultures similar to Bar treatment alone.

### 2.4. Combined MnTBAP/Bar Treatment Ameliorates Maximal Respiration and Spare Respiratory Capacity of HGPS Cells

**Figure 4:**
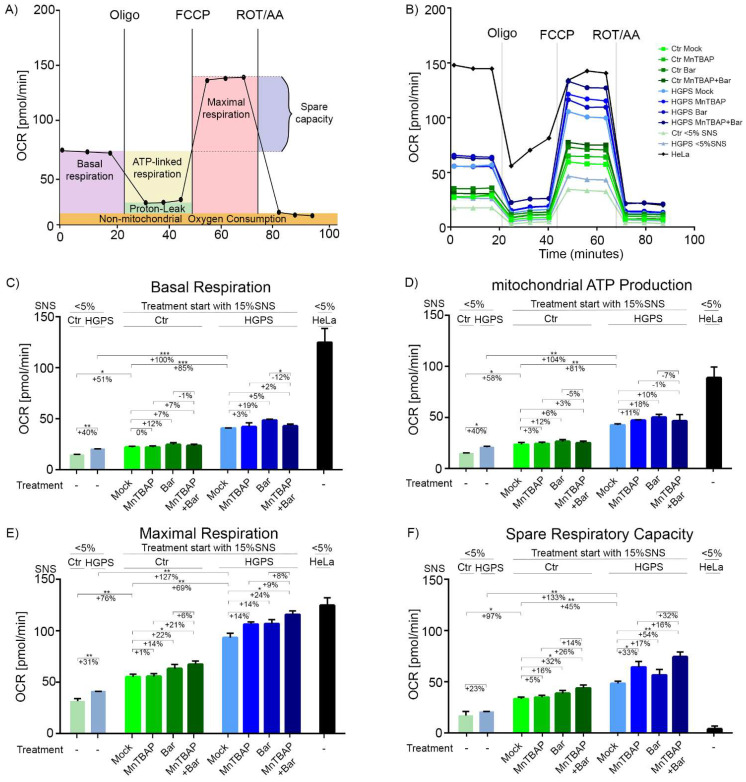
Mitochondrial function is altered in HGPS fibroblasts. (**A**) Schematic overview of the cell mito-stress test profile created using Inkscape. (**B**–**F**) Oxygen-consumption rates (OCR), determined using Seahorse XF96 Flux analyzer after cells’ treatment with a mock solution, 5 µM MnTBAP, 1 µM Bar or the MnTBAP/Bar combination for 9 d. Additional parameters, such as mitochondrial ATP production (**D**), maximal respiration (**E**), and spare respiratory capacity (**F**), were calculated using Wave Software (Agilent Technologies, V. 2.6.1.53). Graphs show the mean ± SD (*n* = 3; * *p* < 0.05, ** *p* < 0.01, *** *p* < 0.001).

Mitochondrial dysfunction in HGPS cells has been reported [28,45,60], but the cellular mechanisms underlying this defect are largely unknown. To investigate the possible effects of MnTBAP/Bar treatment on the dynamics of mitochondrial function and energy metabolism, we performed a respirometric assay. Respirometry is the gold standard for measuring the mitochondrial oxidative function [61]. It reflects the activity of the electron transport chain (ETC) complexes, which are essential for energy production and are required for mitochondrion-dependent cell metabolism [61]. We determined the oxygen-consumption rate (OCR) and extracellular acidification rate (ECAR) in HGPS and control cells. Figure 4A shows a schematic representation of the various readouts that can be derived from the cell mito-stress profiling used in this study. To reveal the key parameters of mitochondrial functions, we used the following respiration modulators for assaying the live cells: oligomycin, an inhibitor of the ATP synthase (complex V) [62]; FCCP, an uncoupler disrupting the proton gradient [63]; and rotenone A, a complex I inhibitor with antimycin A, a complex III inhibitor [64,65]. After measurement of the basal respiration rate, oligomycin, FCCP, and rotenone with antimycin A were sequentially added to determine the ATP production, maximal respiration, and non-mitochondrial respiration of cells, respectively (Figure 4B–E). The obtained values allowed further estimations of proton leakage and the spare respiratory capacity of the cells (Figure 4F and Figure S4). To determine changes in mitochondrial functional upon the different regimens, we measured the OCR of control and HGPS cells from early (<5% SNS, Ctr: passage ≤ 21; HGPS: passage ≤ 19) and late passages (approximately 15% SNS, Ctr: passage ≥ 27; HGPS: passage ≥ 24) (Figure 4B,C). In the experiment, we also included the cancer cell line, HeLa, as a control cell line that is glycolysis-dependent [66].

Young HGPS cells (cultures at <5% SNS) showed higher basal respiration rates than control cells, indicating that HGPS cells require a higher OxPhos activity to meet the basal energy demands (Figure 4B,C). In addition, increased basal respiration was observed in both HGPS and control cells from late passages (cultures at ~15% SNS), although the rate was higher in HGPS than in control cells (Figure 4B,C). These observations indicated that basal respiration increased with replicative senescence in both cell types, and were in agreement with previous studies [67]. Our data also implied that the increased basal respiration in HGPS cells was associated with progerin expression and processes inherent to senescence. Comparing HGPS cells with control cells at the same SNS status (5% SNS or ~15% SNS), we observed that HGPS cells produce more ATP via OxPhos than control cells, whereas proton leakage was not affected in either cell type regardless of senescence status (Figure 4B,D and Figure S4). Young HGPS fibroblasts displayed increased maximal respiration compared to the young control cells (5% SNS) (Figure 4B,E). The maximal respiration rate was increased in both control and HGPS cells during replicative senescence but remained higher in HGPS cells than in controls (~15% SNS) (Figure 4B,E). The spare respiratory capacity is an important indicator of a cell’s ability to respond to energy demand under stress conditions [68]. Spare respiratory capacity was increased during replicative senescence in both control and HGPS cells and was consistently higher in HGPS cells than in controls (Figure 4B,F). These findings suggest that HGPS cells develop an adaptive stress response because of progerin expression and changes related to cellular senescence. Collectively, these data indicated that OxPhos is increased in HGPS cells.

Next, we determined the effect of MnTBAP, Bar, and their combination on control and HGPS cell energetic profiles (Figure 4B–F and Figure S4). The same trend was apparent in the treated control and HGPS cells. MnTBAP did not affect OxPhos, whereas Bar increased the OCR without being significant (Figure 4B,C). Oxygen consumption was slightly increased in cells treated with the MnTBAP/Bar combination, but this increase was not significant (Figure 4B,C). All treatment regimens slightly increased mitochondrial ATP production in both cell types. Proton leakage was not affected by the treatments (Figure S4). In addition, MnTBAP and Bar treatments increased the maximal respiration in HGPS cells. The combined MnTBAP/Bar treatment induced a further increase in maximum respiration (Ctr: +22%; HGPS: +24%) (Figure 4B,E). The results indicated a positive effect of MnTBAP with Bar on maximum respiration. Furthermore, MnTBAP increased spare respiratory capacity in both control (statistically not significant) and HGPS (statistically significant) cells. A similar trend was also observed upon the Bar treatment (Figure 4B,F). The drug combination showed a positive effect on spare respiratory capacity in both cell types (Ctr: +32%; HGPS: +54%). Collectively, these findings indicate an increased spare respiration capacity in cells treated with MnTBAP/Bar, which may allow the cells to adapt more rapidly to increased energy demands under stress conditions.

**Figure 5:**
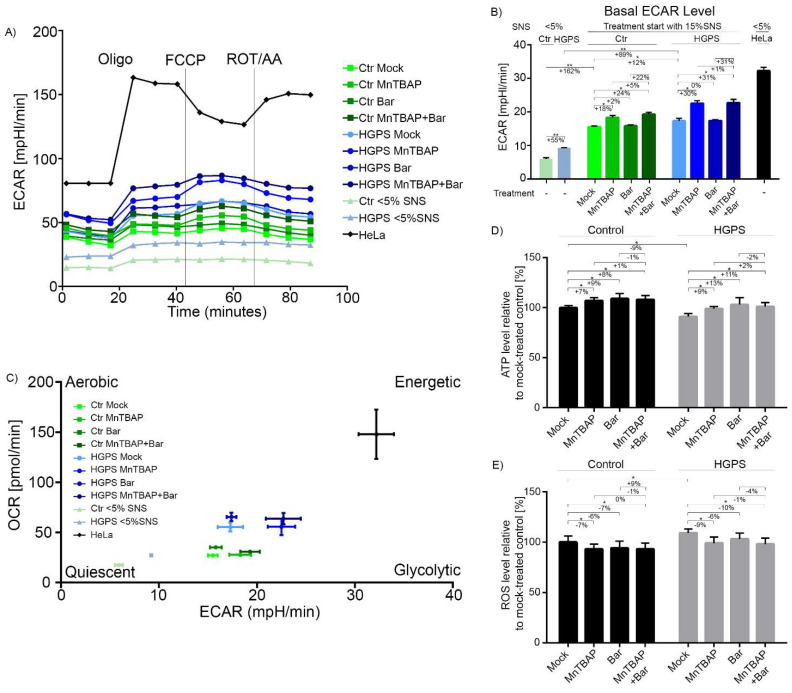
Effect of combination treatments on glycolysis, ATP, and ROS levels. (**A**–**E**) Extracellular acidification (ECAR) was determined using a Seahorse XF96 Flux analyzer after cells were treatet with a mock solution, 5 µM MnTBAP, 1 µM Bar, or the MnTBAP/Bar combination for 9 d. (**C**) Energy phenotype profile displayed as a scatter plot of OCR and ECAR. (**D**) Intracellular ATP levels were measured using a CellTiter-Glo luminescence ATP assay. (**E**) Intracellular ROS levels were determined based on oxidized dichlorofluorescein (DCF) levels measured using DCFDA Cellular Detection Assay. Graphs show the mean ± SD (*n* = 3; * *p* < 0.05, ** *p* < 0.01).

To determine the impact of MnTBAP and Bar on glycolysis, we measured the basal ECAR levels (Figure 5). 

The basal ECAR levels were already higher in young HGPS cells (<5% SNS) compared to young controls (Figure 5A,B). While basal ECAR levels increased during replicative senescence (~15% SNS) in both cell types, they remained higher in HGPS cells than in controls (Figure 5A,B). Our results are in accordance with previous studies, i.e., that glycolysis is elevated in senescent cells [67]. The MnTBAP, Bar, and MnTBAP/Bar treatments resulted in similar glycolytic trends in both cell types (Figure 5A,B). While Bar did not affect ECAR, MnTBAP and MnTBAP/Bar increased glycolysis in both cell types (Figure 5A,B). Collectively, glycolysis was increased during replicative senescence in both control and HGPS, and it was further increased by MnTBAP treatment. 

Together, HGPS cells showed increased OxPhos and glycolysis compared with control cells (Figure 5C). The basal respiration and ECAR of HGPS cells increased during senescence. Upon MnTBAP treatment, glycolysis increased, while oxygen consumption was not affected (Figure 5C). The Bar treatment increased oxygen consumption (statistically not significant) without affecting glycolysis, and the drug combination increased oxygen consumption (statistically not significant) and glycolysis (statistically significant) (Figure 5C). Overall, the combined drug treatment enhanced the maximal respiration and the spare respiratory capacity in both control and HGPS fibroblasts.

In addition, we determined the intracellular ATP levels in control and HGPS cells at 15% SNS. In agreement with the OCR data, the ATP levels in HGPS cells appeared 9% lower than those in control cells (Figure 5D). This indicated that even though HGPS cells showed elevated OxPhos, compared with control cells, this was insufficient to allow them to reach the ATP levels of control cells. All treatment regimens slightly increased the ATP levels in both cell types (Figure 5D).

Because MnTBAP is a well-characterized antioxidant [46], we tested its ability to reduce ROS levels in HGPS cells. We determined ROS levels in control and HGPS cultures (15% SNS) after 9 d of treatment with the different regimens (Figure 5E). Basal ROS levels in HGPS cells were 9% higher than those in control cells (Figure 5E). As anticipated, the MnTBAP treatment reduced the ROS levels in both control and HGPS fibroblasts (Ctr: −7%; HGPS: −9%) (Figure 5E). The Bar treatment also reduced the ROS levels, but this effect was not significant. The combined MnTBAP/ Bar treatment reduced the ROS levels to levels similar to those observed with MnTBAP alone (Ctr: −7%, HGPS: −10%) (Figure 5E). 

### 2.5. Combined MnTBAP/Bar Treatment Reduces DNA Damage in HGPS Fibroblasts

**Figure 6:**
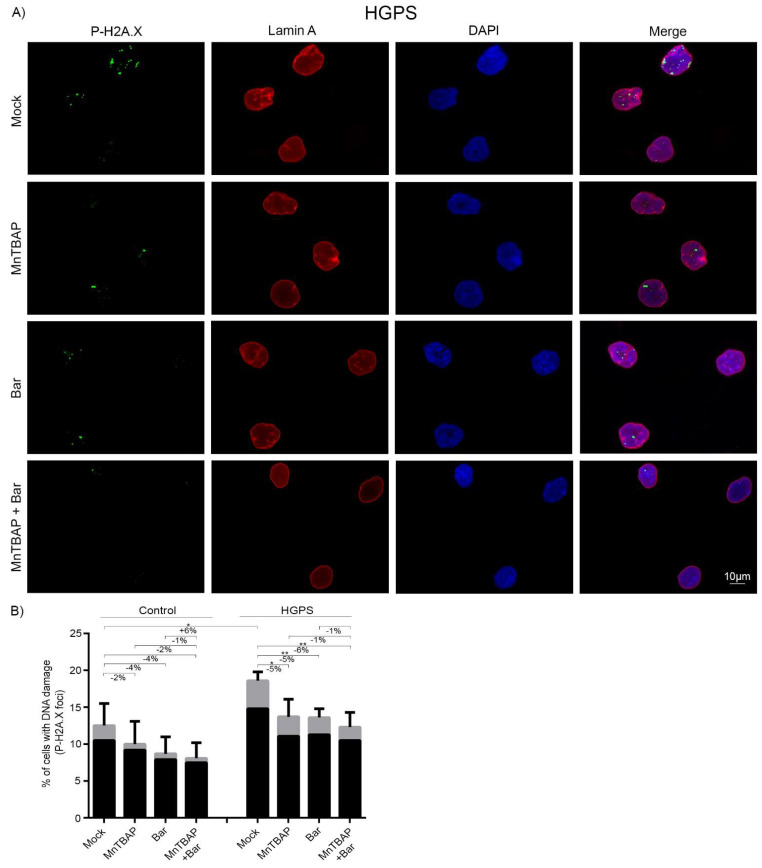
MnTBAP/Bar combination treatment reduces the number of p-H2A.X foci. (**A**) Representative immunofluorescence images of HGPS fibroblasts after 9 d with a mock solution, 5 µM MnTBAP, 1 µM Bar, or the MnTBAP/Bar combination. The cells were stained with anti-phospho histone H2A.X (Ser139) (green) and anti-lamin A (red) antibodies and counterstained with DAPI. (**B**) The number of nuclei with low DNA damage (black) or severe DNA damage (grey) after treatment was scored in at least 900 nuclei for each condition. Graphs show the mean ± SD (*n* = 4; * *p* < 0.05, ** *p* < 0.01).

The levels of DNA damage in HGPS cells are high because of altered DNA repair response and genomic instability in the cells [69]. Previous studies have demonstrated that ROS levels are increased in HGPS cells [10], and it has been established that high ROS levels can induce DNA damage [43,70]. Therefore, we next evaluated whether the MnTBAP, Bar, or MnTBAP/Bar treatments could reduce the amount of DNA damage in HGPS cells (Figure 6A,B). 

To this end, control and HGPS cultures (15% SNS) were treated for 9 d with the different regimens. The cells were then stained with an antibody against γ-H2A.X, a marker of DNA damage (Figure 6A and Figure S5). We also scored the total number of nuclei containing DNA damage foci and determined the number of nuclei with 1-5 γ-H2A.X foci (Figure 6B) and those with more than 5 γ-H2A.X foci (Figure 6B). As previously reported, HGPS cells showed higher levels of DNA damage than control cells [69,71] (Figure 6B). The incidence of nuclei with severe DNA damage, as indicated by the percentage of nuclei exhibiting more than 5 γ-H2A.X foci, was also higher in HGPS cells than in the control cells (Figure 6B). After all treatments, the number of γ-H2A.X foci was reduced in control cells, but this effect was not significant. Nonetheless, the MnTBAP/Bar treatment reduced the extent of DNA damage levels in HGPS cells, in a similar proportion to each drug alone (Figure 6B). Collectively, these findings indicated that MnTBAP/Bar reduces the amount of DNA damage in HGPS cells.

## 3. Discussion

Patients suffering from HGPS, a rare premature aging disease, have a short life expectancy and develop various age-related conditions [1]. In the current study, we asked whether a combined MnTBAP/Bar treatment could reverse the HGPS cellular phenotype. We showed that the combined MnTBAP/Bar treatment alleviated hallmarks of HGPS, such as premature senescence, nuclear shape, mitochondrial dysfunction, and DNA damage levels. These observations may inform a novel treatment for HGPS.

To date, only one drug, lonafarnib, has been approved by the FDA for the treatment of HGPS [21]. Lonafarnib, an FTI, improves lifespan and bone mineral density, and decreases skeletal rigidity in individuals with HGPS, but it is not a cure [8,19]. It is of note that some HGPS pathologies overlap with those of physiological aging, including alopecia, bone abnormalities, chronic inflammation, and mitochondrial dysfunction [1,13,28]. In this study, we evaluated a combination of drugs that affect different cellular processes, to target various cellular defects in HGPS. MnTBAP is a SOD mimetic that scavenges ROS such as O_2_^−^, and H_2_O_2_ in the mitochondrion, and protects against oxidative stress [46,47]. By contrast, the JAK1/2 inhibitor Bar targets inflammation [12]. We are the first to test a JAK1/2 inhibitor in combination with a SOD mimetic in HGPS fibroblasts to target the mitochondrial dysfunction and reduce chronic inflammation in HGPS.

We used HGPS and control fibroblast cultures (at <5% SNS and ~15% SNS) to determine metabolic alterations that occur in HGPS cells and those occurring during replicative senescence. HGPS cells rapidly enter premature senescence; therefore, we monitored the cultures not according to the passage numbers but according to the senescence index of cultures [12]. This approach permitted us to distinguish between defects induced by the expression of progerin from processes related to cellular aging.

Recent studies have demonstrated a change in the regulation of glycolysis and the oxygen-consumption rates in both HGPS mouse and human fibroblasts [26,60,67,72]. Consequently, we measured the oxygen-consumption rate and the extracellular acidification rates to monitor the oxidative phosphorylation and glycolysis, respectively, in the control and HGPS fibroblasts. The oxygen-consumption and glycolysis levels in HGPS cells were higher than those in the control cells at the same senescence index (<5% SNS and ~15% SNS). This indicates increased OxPhos and glycolysis levels in HGPS cells compared with the control cells at young passages. This finding is in agreement with in vivo studies showing an increased basal respiration in the HGPS mouse model [60]. Together, these observations support the hypothesis that progerin expression triggers a metabolic reprogramming of HGPS cells and is distinct from processes inherent to replicative senescence. The observation of increased OxPhos and glycolysis in HGPS cells could be explained by aging-related mechanisms as follows. Misfolded proteins accumulate during aging [73] and their degradation requires a substantial amount of energy [74]. Because HGPS cells exhibit premature aging, they develop adaptation mechanisms to increase their OxPhos and glycolysis to compensate for other alterations in cellular processes, such as degradation of misfolded proteins, causing the accumulation of progerin.

In this study, we also analyzed the effects of MnTBAP and Bar on cellular metabolism. We showed that the combined MnTBAP/Bar treatment increased the maximum respiration and the spare respiratory capacity of HGPS and control cells. As a result of an increased maximum capacity, the cells are able to rapidly oxidize metabolic substrates to meet their metabolic needs [75]. Spare respiration capacity is the difference between basal respiration and maximal respiration, and our observations reflect an increased cellular capacity to respond to an increased energy demand or stress conditions [68]. This suggests that the combined-drug MnTBAP/Bar treatment increases the cellular respiratory rates under stress, allowing the cells to meet their energy demands. 

Mitochondrial dysfunction and chronic inflammation develop during normal and premature aging [28,76]. In recent years, inflamm-aging (age-related inflammations) has become an important research focus [77]. Two major inflammation pathways are associated with HGPS: the NF-κB pathway and the JAK/STAT signaling pathway [12,78]. We here demonstrated that the combination of MnTBAP with Bar efficiently blocks the activation of the JAK/STAT signaling pathway in HGPS cells; this effect is caused by Bar. We also showed that the combination treatment reduces the expression of pro-inflammatory cytokines, such as CCL2, IL-6, and IL-8, in HGPS cells. This further confirms our assumption that the MnTBAP/Bar drug combination can reduce inflammation in HGPS cells. Pro-inflammatory cytokines, such as IL-6 and IL-8, are secreted by senescent cells and are part of the senescence-associated secretory phenotype [76,79]. Literature shows that MnTBAP can have an impact on immune regulation [80,81]. This influence of MnTBAP on the immune response in combination with the immune modulator Bar could contribute to an additional improvement of chronic inflammation in HGPS. Taken together, the MnTBAP/Bar treatment lowers the pro-inflammatory status and delays cell senescence, which are both hallmarks of aging that lead to tissue and organ damage if left untreated.

Genomic instability is another characteristic of cellular aging [57]. HGPS cells exhibit high levels of DNA damage because of genomic instability and altered DNA repair responses [69]. The stability and integrity of cellular DNA are constantly challenged by various factors, such as exogenous physical and chemical agents, DNA replication errors, and ROS [57]. Elevated ROS levels, as is the case with HGPS, lead to increased DNA damage [10,70]. Remarkably, the combined MnTBAP/Bar treatment reduces ROS levels and thereby reduces DNA damage in HGPS cells. 

The accumulation of progerin at the nuclear envelope in HGPS cells triggers the development of several nuclear abnormalities, including altered nuclear shape and blebbing [3,9]. We determined that the MnTBAP/Bar treatment reduced progerin levels by boosting autophagy in cells. Consistently with these observations, it has been reported that progerin can be degraded via autophagy [12,82]. Concomitantly with reduced progerin levels, the number of dysmorphic nuclei was also decreased in HGPS fibroblasts after the combination treatment. Taken together, these results indicate that the MnTBAP/Bar treatment activates autophagy, and thereby enhances progerin clearance, which ameliorates HGPS nuclear abnormalities.

In summary, the combined MnTBAP/Bar treatment reversed the cellular HGPS phenotypes, particularly improving mitochondrial function, with an increased spare respiratory capacity and intracellular ATP levels. The combined treatment efficiently reduced progerin levels, ameliorated aberrant nuclear morphology, delayed senescence, and reduced the senescence-associated inflammatory phenotype. By ameliorating HGPS defects on the cellular level, the combination of the JAK inhibitor Bar and the SOD mimetic MnTBAP is also expected to induce similar beneficial effects on the organism level. This should be investigated further in vivo in an HGPS mouse model. To date, lonafarnib is the only treatment for individuals with HGPS. Unfortunately, lonafarnib alone does not alleviate all the symptoms that affect patients with HGPS, and other therapeutic strategies, including various drug combinations, should be investigated. The current study shows that the combined MnTBAP/Bar treatment delays cellular senescence and inflammation, which are important drivers of premature aging and age-related conditions, which develop rapidly in patients with HGPS. Hence, this treatment could potentially benefit patients with HGPS as well as individuals suffering from various age-related pathologies.

## 4. Materials and Methods

### 4.1. Cell Culture

Fibroblast cell lines obtained from HGPS patients HGADFN003 (2-year-old male), and HGADFN127 (3-year-old female) were all obtained from the Progeria Research Foundation Cell and Tissue Bank (http://www.progeriaresearch.org, accessed on 3 April 2020). Control fibroblasts were obtained from the Coriell Institute for Medical Research (Camden, NJ, USA). GMO1651C (13-year-old female), and GM01652C (11-year-old female) were used. The cells were cultured in Dulbecco’s Modified Eagle medium+ GlutaMAX^TM^ (high glucose DMEM, Gibco^TM^, Thermo Fisher Scientific Inc., Waltham, MA, USA, 31966047) supplemented with 15% fetal bovine serum (FBS, Gibco^TM^, Thermo Fisher Scientific Inc., Waltham, MA, USA, 10270106), 1% l-glutamine (200 mM, Thermo Fisher Scientific Inc., Waltham, MA, USA, 25030123), 1% penicillin-streptomycin (10,000 U/mL, Gibco^TM^, Thermo Fischer Scientific Inc., Waltham, MA, USA, 15140122), and 0.5% gentamycin (10 mg/mL, Gibco^TM^, Thermo Fischer Scientific Inc., Waltham, MA, USA, 15710049). The cells were subcultured when they reached 80% confluence. All cultures were grown in an incubator (Binder, Tuttlingen, Germany, 9140-0046) with a humidified chamber at 37 °C and 5% CO_2_. For the experiments, we used fibroblast cell lines from two HGPS patients, which we compared with two control cell lines to make a general, comprehensive statement about their ratio. For simplicity of presentation, we refer to these values as HGPS and Control.

### 4.2. Drug Treatment

Fibroblasts were treated with 5 µM MnTBAP chloride (Santa Cruz Biotechnology, Dallas, TX, USA, CAS 55266-18-7), 1 µM Baricitinib (Abcource Diagnostics GmbH, Munich, Germany, LY3009104, INCB028050) or with a combination of the two drugs at above indicated concentrations. The treatment period was 9 days, with the culture medium containing the drugs changed every other day.

### 4.3. Senescence-Associated Beta-Galactosidase Staining (SA-β-Gal) 

Dimri’s staining protocol, was used to detect cellular senescence [83]. Briefly, cells were seeded in a 3.5 cm dish and cultured for at least 72 h. They were washed in Dulbecco’s phosphate-buffered saline (PBS; Sigma-Aldrich, St. Louis, MO, USA, D8537) for 5 min. To fix the cells, a fixation solution containing 0.2% glutaraldehyde solution (Sigma-Aldrich, G5882) and 2% formaldehyde solution (Merck KGaA, Darmstadt, Germany, 104003) was prepared in PBS. After 5 min of fixation, the cells were washed twice with PBS for 5min. Subsequently, the cells were incubated overnight at 37 °C (without CO_2_) in the SA-β-Gal staining solution, which contained 5 mM potassium ferricyanide (III) (KGaA, 104973), 5 mM potassium ferrocyanide (II) (Sigma-Aldrich, St. Louis, MO, USA, P9387), 2 mM MgCl_2_ (Sigma-Aldrich, M1028), 150 mM NaCl (Sigma-Aldrich, St. Louis, MO, USA, M1028), 0.5 mg/mL 5-bromo-4-chloro-3-indolyl-β-D-galactopyranoside (X-gal; Roche, Basel, Germany, 3117073001), and 40 mM citrate/sodium phosphate buffer (Sigma-Aldrich, St. Louis, MO, USA, S5136), pH = 6. For the analysis, 1000 cells were counted from each sample with a Axiovert 40 CFL bright field microscope. The senescence test was performed at each passage and before the experiments as well as after seeding in some experiments to ensure that the cultures exhibited the same percentage of senescent cells to allow comparison. 

### 4.4. Western Blot Analysis

Cells were harvested by scraping or trypsinization. To estimate the protein concentration, a Bradford assay was used, with BSA as a standard (BioRad Laboratories, Hercules, CA, USA, 5000206). After electrophoresis, the proteins were transferred onto nitrocellulose membranes, which then were blocked in 5% non-fat milk for 1 h, and incubated overnight at 4 °C with the following antibodies: anti-lamin A/C (E1, sc-376248, Santa Cruz Biotechnology, Dallas, TX, USA, dilution 1:10,000), anti-P-STAT1 (9167S, TYR701, Cell Signaling, dilution 1:1000), STAT1 (14994, D1K9Y, Cell Signaling, Danvers, MA, USA, dilution 1:1000), anti-P-STAT3 (9145S, THR705, Cell Signaling, Danvers, MA, USA, dilution 1:1000), anti-STAT3 (9139, 12H6, Cell Signaling, Danvers, MA, USA, dilution 1:1000), and anti-β-Actin (A1978, Sigma-Aldrich, St. Louis, MO, USA, dilution 1:10,000). The membrane was then washed three times with TBS-T for 5min. Next, the membrane was incubated with the corresponding secondary antibody conjugated with horseradish peroxidase (Jackson ImmunoResearch Laboratories, Westgrove, PA, USA). ChemiDoc^TM^ MP was used to visualize the signals, which were quantified by densitometry using ImageJ software (NIH). Images were analyzed with Fiji [84], and the signals were normalized to that of β-Actin.

### 4.5. Cell Number Determination

To determine the cell number, 1.5 × 10^5^ cells were seeded in 10 cm dishes and allowed to adhere overnight. The following day, cells were treated with drugs as described in Section 2.2. After 9 days of treatment, cells were trypsinized and counted using Muse^TM^ Cell Analyzer (Merck KGaA, Darmstadt, Germany). For each further experiment where a certain cell count was required, we counted the cells using the Muse^TM^ Cell Analyzer (Merck KGaA, Darmstadt, Germany). 

### 4.6. Respirometic Assay

The oxygen-consumption rate (OCR) and the extracellular acidification rate (ECAR) in fibroblasts were measured using the Seahorse FX96 Extracellular Analyzer (Agilent Technologies). The cells were analyzed using a Mito-Stress Test Kit, according to the manufacturer’s protocol. For experiments, 1.5 × 10^4^ cells were seeded in triplicate into a 96-well plate. To prepare the Seahorse XF DMEM assay medium, 10 mM glucose, 1 mM pyruvate, and 2 mM L-glutamine were added to the medium. The final concentrations of 2.5 µM oligomycin, 2 µM carbonyl cyanide-4 (trifluoromethoxy) phenylhydrazone (FCCP), and 1 µM rotenone/antimycin A were used. Port A of the flux cartridge was loaded with 20 µL oligomycin; port B was loaded with 22 µL FCCP, and 25 µL of rotenone/antimycin A was added into port C. The assay was performed in triplicates in three independent experiments.

### 4.7. Autophagy Assay

Autophagy was determined using a Cayman Chemicals Autophagy/Cytotoxicity Dual Staining Kit (Cayman Chemicals, Ann Arbor, ML, USA, 600140). The kit contains the fluorescent compound monodansylcadaverine (MDC), which is incorporated into multilamellar bodies and used for the detection of autophagic vacuoles in cells. For experiments, an equal number of 1.5 × 10^5^ cells were seeded in culture plates and treated with mock, single drug, or a drug combination treatment for 8 days as described in Section 4.2. On day 8, 20,000 cells were seeded in 96-well plates with medium containing mock or the indicated drug treatment and allowed to adhere overnight. The next day, after 9 days of drug treatment MDC was added at a ratio of 1:1000. A FLUOstar Omega microplate reader (BMG Labtech, Ortenberg, Germany) was used to measure the fluorescence of autophagic vacuoles (Ex: 355 nm; Em: 520 nm). All measurements were repeated in at least three independent experiments.

### 4.8. ROS Measurements

The 2′,7′-Dichlorofluorescein Diacetate (DCFDA) Cellular ROS Detection Assay Kit (Abcam, Cambridge, UK, ab113851) was used for the determination of ROS levels. A total of 1.5 × 10^5^ control and HGPS fibroblasts were seeded in parallel in culture plates and were treated with the drug regimens as described in Section 4.2. After 8 days of treatment, 2.0 × 10^4^ cells were transferred into 96-well plates with the indicated drug treatment and left attached overnight. After 9 days of drug treatment, the cells were incubated with 25 µM DCFDA for 45 min at 37 °C. The cell-permeating reagent DCFDA diffuses into the cell where it is deacetylated by esterases and then reacts with intracellular ROS to form the fluorescent compound DCF. Accordingly, DCF was detected by using FLUOstar Omega microplate reader (BMG Labtech, Ortenberg, Germany, Ex: 485 nm; Em: 520 nm). All measurements were repeated in at least three independent experiments.

### 4.9. Immunohistochemistry

Drug-treated cells were seeded on ethanol-cleaned coverslips and were incubated at least overnight to attach. Subsequently, the cells were fixed with 4% PFA for 10 min at RT followed by permeabilization with 0.2% Triton-100 in PBS for 10 min at RT. Following this, the cells were blocked with 10% FBS in PBS for 30min at RT. Primary antibodies were diluted in PBS with 10% FBS. The following primary antibodies were used: anti-progerin [39], anti-lamin A (L1293-200UL, Sigma-Aldrich, St. Louis, MO, USA, dilution 1:2000), anti-lamin A/C (E1, sc-376248, Santa Cruz Biotechnology, Dallas, TX, USA, dilution 1:2000), anti-phospho-histone H2A.X Ser139 (05-636, clone JBW301, Merck KGaA, Darmstadt, Germany, dilution 1:2000), and anti-p21 (MA5-14949, Invitrogen, Waltham, MA, USA). Next, the samples were washed with PBS and incubated with the corresponding secondary antibodies at RT for 1 h: affinity-purified Alexa Fluor^®^ 488 or 555 conjugated anti-rabbit/mouse antibodies (Life Technologies, Carlsbad, CA, USA). Following this, samples were counterstained with DAPI in a Vectashield mounting medium (Vector). The samples were imaged using an Axio Imager D2 fluorescence microscope (AcxioCam MRm, objective x40 oil, x63 oil, Carl Zeiss, Berlin, Germany). The images were analyzed with Fiji [84] and imported into Adobe Photoshop CC 2017 for presentation.

For the determination of dysmorphic nuclei, cells were stained with anti-lamin A (L1293-200UL, Sigma-Aldrich, St. Louis, MO, USA, dilution 1:2000) as described above. We counted dysmorphic nuclei, assessing parameters such as folding, blebbing, invagination, and abnormal nuclear size. To eliminate variation, an average of 900 nuclei were counted from three experimental replicates. We and others have previously published very detailed descriptions on HGPS nuclear dysmorphy [85]. 

### 4.10. Measurements of Intracellular ATP Levels

To measure the intracellular ATP levels, CellTiter-Glo^®^ Luminescent Cell Viability Assay (Promega, Madison, WI, USA) was used according to the manufacturer’s protocol (Promega, Madison, WI, USA). Briefly, after cell lysis, the amount of ATP in cells was determined by assessing the luminescent signal generated in a luciferase reaction. For the assay, 1.5 × 10^5^ control and HGPS cells were seeded in culture plates and treated with mock, single drug, or drug combination treatment as described in Section 4.2. On day 8, 2.0 × 10^4^ cells were seeded in 96-well plates and cultured overnight with the indicated drug treatment. The next day, the CellTiter-Glo reagent was added to the cells, the samples were incubated for 10 min, and the luminescent intensity was measured using a FLUOstar Omega microplate reader (BMG Labtech, Ortenberg, Germany). All measurements were repeated in at least three independent experiments.

### 4.11. Gene Expression Analysis

Approximately 1.0 × 10^6^ fibroblasts were collected for RNA analysis. RNA was extracted from cell pellets using a GenJET RNA Purification Kit (Thermo Fisher Scientific Inc., Waltham, MA, USA) according to the manufacturer’s protocol. A NanoDrop ND-100 spectrophotometer (Thermo Fisher Scientific Inc., Waltham, MA, USA) was used to assess the RNA quantity and purity. For experiments, 1000 ng of RNA was reverse-transcribed into cDNA using a High-Capacity cDNA Reverse Transcription Kit (Thermo Fisher Scientific Inc., Waltham, MA, USA). Real-time PCR primers were designed using NCBI/Primer-BLAST [86]. All the evaluated genes and their corresponding primers are listed in Table S1. To perform real-time PCR, a PowerUp^TM^ SYBR^TM^ Green Master Mix (Applied Biosystems^TM^, Thermo Fisher Scientific Inc., Waltham, MA, USA) was used. For the experiment, 300 nM of each primer and 50 ng of the template in a 20 µL reaction volume were used for optimal detection. The thermal cycling profile consisted of an initial denaturation step at 95 °C for 20 s, followed by 45 cycles of 95 °C for 3 s and 60 °C for 30 s. All amplification signals were observed between cycles 10 and 40. All experiments were repeated at least three times. GAPDH expression was used as an internal control. The thermal cycler StepOnePlus^TM^ Real-Time PCR System was used.

### 4.12. Toxicity Assay

To ensure that the drug concentration of MnTBAP is not cytotoxic for control and HGPS fibroblasts, we tested different concentrations of MnTBAP on the cells. Therefore, in a 10 cm dish 1.5 × 10^5^ cells were seeded and allowed to attach overnight. The next day, cells were treated with different MnTBAP concentrations (0–50 µM) for 4 days. The culture media containing the drug were changed every other day. After 4 days, the cells were trypsinized and counted using a Muse^TM^ Cell Analyzer (Merck KGaA, Darmstadt, Germany). The treatment period of 4 days was considered sufficient to detect cellular toxicity and determine MnTBAP concentration that maintains cell growth in similar range as mock-treated cultures.

For the other experiments, a different treatment period was chosen because we previously demonstrated that the levels of autophagy start to increase at day 4 and the maximum increase is observed by day 9 [12]. Hence, cellular senescence is known to develop slowly and requires more than 5 days, as described in the literature [87]. Consequently, a treatment period of 9 days allowed us to investigate all the various parameters analyzed in this study and perform a rigorous comparison.

### 4.13. Statistical Analysis

Comparison of the distinctive characteristics of HGPS fibroblasts and healthy control fibroblasts (treated and untreated) was performed using Student’s t test, and a one-way analysis of variance (ANOVA) was used for multiple comparison (*n* ≥ 3, * *p* < 0.05, ** *p* < 0.01, *** *p* < 0.001); *p* < 0.05 was considered to indicate statistical significance. Data are presented as the mean ± standard deviation (SD). GraphPad Prism (version 6.01) was used for all statistical analyses. Schematics were created using Inkscape.

## 5. Conclusions

Taken together, the combination of MnTBAP and Bar improved the spare respiratory capacity and the maximal respiration in HGPS fibroblast after 9 days of treatment. Hence, this combination treatment also delayed senescence and enhanced autophagy. However, MnTBAP/Bar treatment decreased inflammatory cytokines, progerin levels, nuclear dysmorphism, and DNA damage to levels comparable to Bar treatment alone.

## Data Availability

Data are contained within the article and Supplementary Materials.

## Supplementary Materials

The following supporting information can be downloaded at: https://www.mdpi.com/article/10.3390/ph15080945/s1, Figure S1: Cell cytotoxicity of MnTBAP at different concentrations; Figure S2: Anit-p21-immunostaining in control fibroblasts after treatment with the different regi-mens; Figure S3: Progerin immunostaining in control fibroblasts after the different regimens; Figure S4: Proton leakage measurements; Figure S5: p-H2A.X immunostaining in control fibroblasts after treatments; Figure S6: Full-length scan of Western blots from Figures 2A,B and 3A; Table S1: Primers used for real-time PCR quantitative PCR analysis.
