# Supplementary material for: Impact of MnTBAP and Baricitinib Treatment on Hutchinson–Gilford Progeria Fibroblasts
Source: Pharmaceuticals (Basel). 2022 Jul 29;15(8):945. doi: 10.3390/ph15080945 (PMC9415676; doi:10.3390/ph15080945)
Supplement: Supplementary file 1 [file pharmaceuticals-15-00945-s001.zip › pharmaceuticals-1740974-supplementary.pdf]

pharmaceuticals

Supplementary Materials

Impact of MnTBAP and Baricitinib treatment on Hutchinson-Gilford progeria fibroblasts

Elena Vehns [1], Rouven Arnold [1] and Karima Djabali [1,*]

[1] Epigenetics of Aging, Department of Dermatology and Allergy, TUM school of Medicine, Munich Institute of Biomedical Engineering, Technical University of Munich (TUM), 85748 Garching, Germany 1; djabali@tum.de
* Correspondence: djabali@tum.de ; Tel.: ++049-089-289-10920

Supplementary Materials.

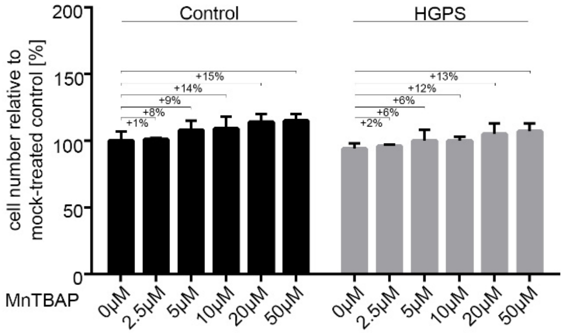

Figure S1. Cell cytotoxicity of MnTBAP at different concentrations. The cell numbers of control and HGPS cells after 4 days treatment with a mock and different concentrations of MnTBAP (2.5µm – 50µm) (n =3).

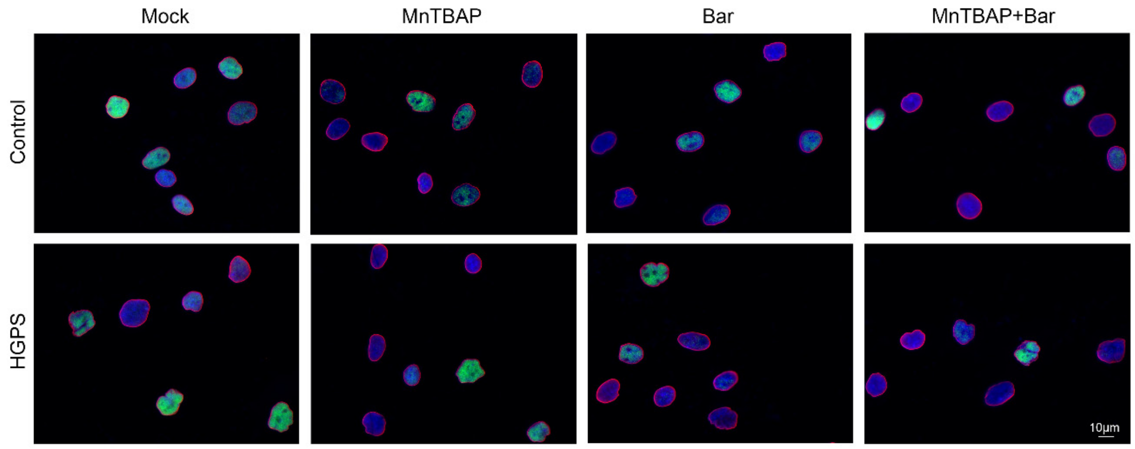

Figure S2. Anit-p21-immunostaining in control fibroblasts after treatment with the different regimens. Representative immunofluorescence images of control fibroblasts after 9 d of treatment with a mock solution, 5 µM MnTBAP, 1 µM Bar or the MnTBAP/Bar combination. The cells were stained with anti-p21 (green) and anti-lamin A/C (red) antibodies and counterstained with DAPI. At least 900 nuclei were counted for each condition. (scale bar 10µm, n=4).

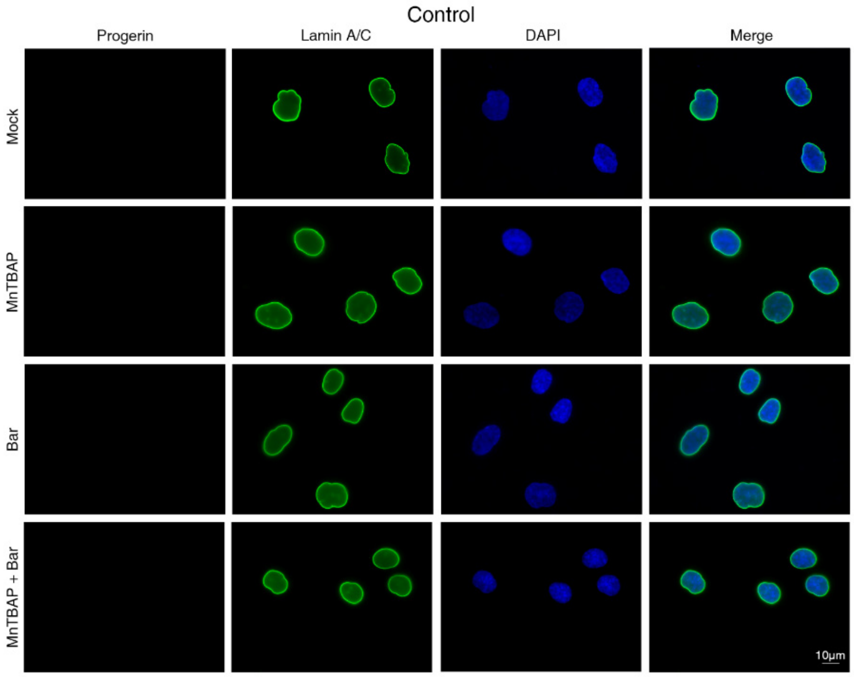

Figure S3. Progerin immunostaining in control fibroblasts after the different regimens. Representative immunofluorescence images of control fibroblasts after 9 d of treatment with a mock solution, 5 µM MnTBAP, 1 µM Bar or the MnTBAP/Bar combination. The cells were stained with anti-progerin (red) and anti-lamin A/C (green) antibodies and counterstained with DAPI (scale bar 10µm, n=4).

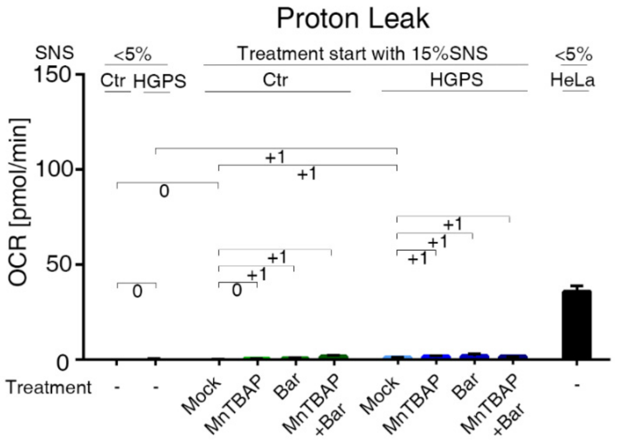

Figure S4. Proton leakage measurements. Proton leakage determined using Seahorse XF96 Flux analyzer after treatment of the control and HGPS cells with mock, 5 µM MnTBAP, 1 µM Bar or with the MnTBAP/Bar combination for 9 d, (n=3).

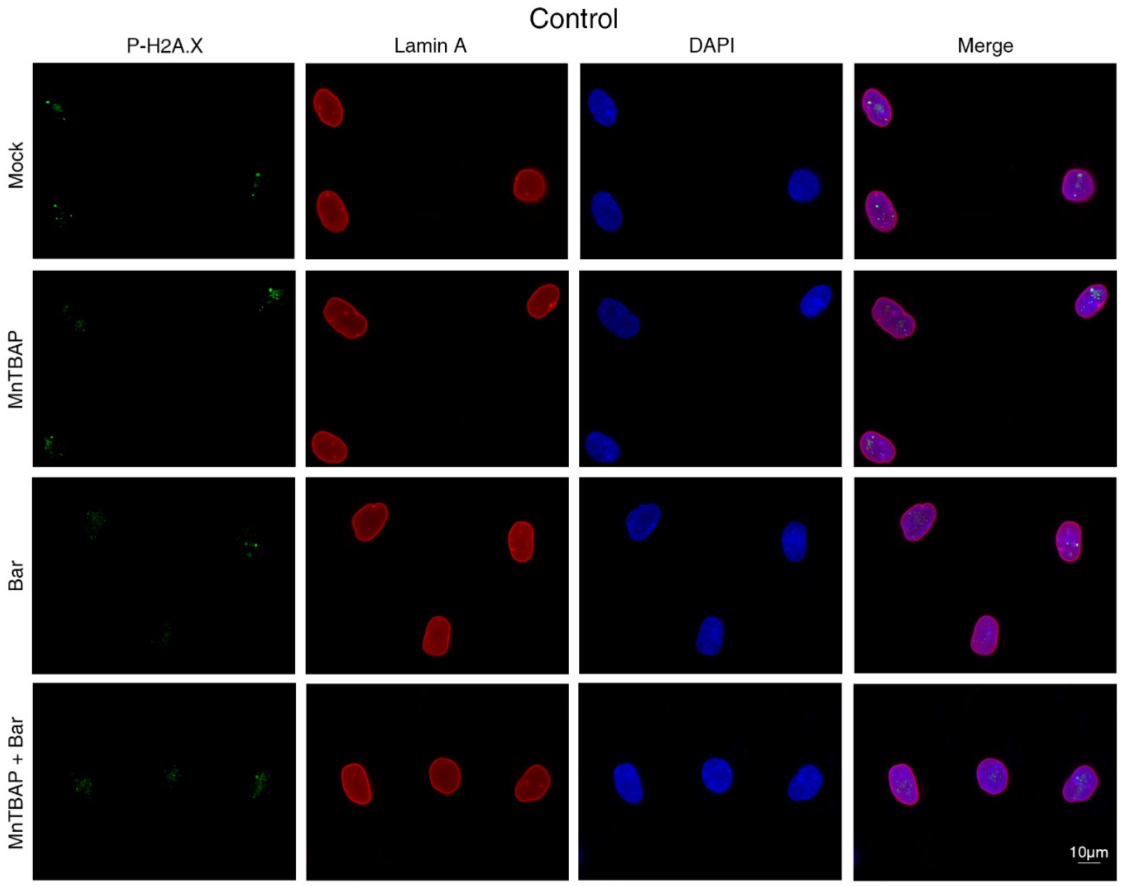

Figure S5. p-H2A.X immunostaining in control fibroblasts after treatments. Representative immunofluorescence images of control fibroblasts after 9 d of treatment with a mock solution, 5 µM MnTBAP, 1 µM Bar or the MnTBAP/Bar combination. The cells were stained with anti-p-Histone H2A.X (Ser139) (green) and anti-lamin A (red) antibodies and counterstained with DAPI (scale bar 10µm, n=4).

Full-length scan of western blot in Figure 2A

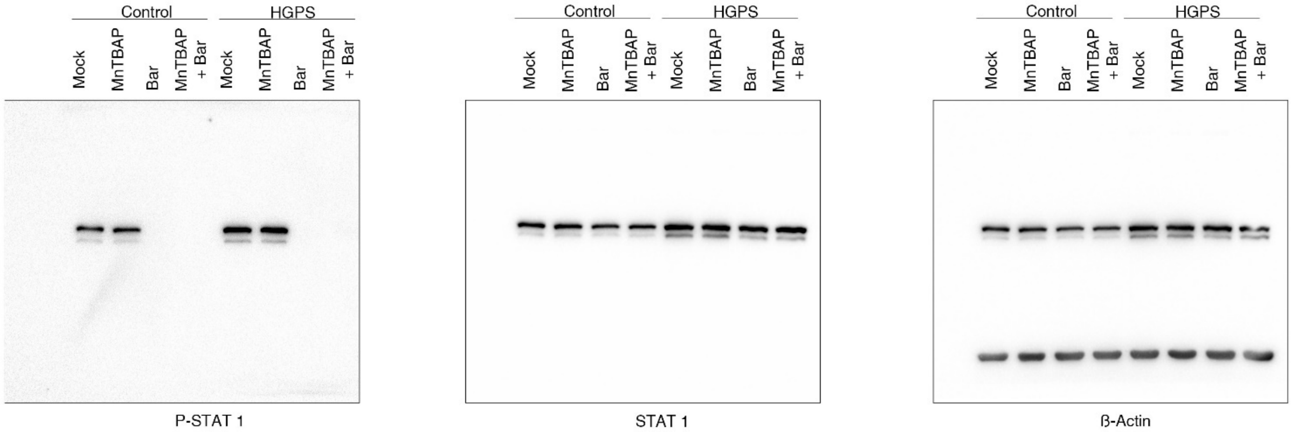

Full-length scan of western blot in Figure 2B

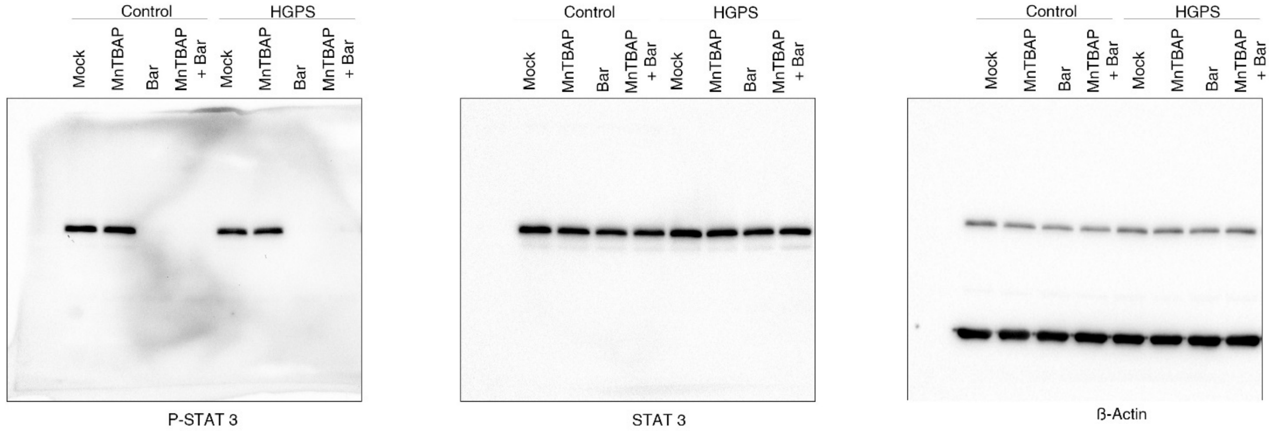

Full-length scan of western blot in Figure 3A

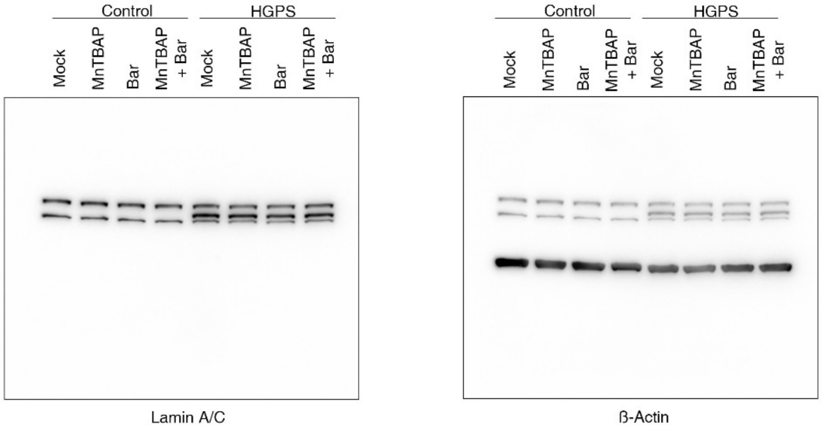

Figure S6. Full-length scan of western blots from Figure 2A, 2B, Figure 3A.

Primer sequence	Target Gene	Product size (bp)	Melting Temp	Info
FW:5'-CGCCAATGACTCAGAGGAAGA-3'<br>REV:5'-AGGGCGTCATTCAGGATGAA-3'	IL-1	120	59.79 ℃<br>59.09 ℃	doi.org/10.1371/journal.pone.0002301
FW:5'-AGCATGAAAGTCTCTGCCGC-3'<br>REV:5'-GGCATTGATTGCATCTGGCTG-3'	CCL2	93	62.9 ℃<br>66.0 ℃	doi.org/10.1172/jci.insight.87023
FW:5'-GGTACATCCTCGACGGCATCT-3'<br>REV:5'-GTGCCTCTTTGCTGCTTTCAC-3'	IL-6	81	63.6 ℃<br>62.4 ℃	doi.org/10.1152/ajpendo.00255.2002
FW:5'-CTGGCCGTGGCTCTCTTG-3'<br>REV:5'-CCTTGGCAAAACTGCACCTT-3'	IL-8	69	63.2 ℃<br>62.5 ℃	doi.org/10.1172/jci.insight.87023

Table S1. Primers used for real-time quantitative PCR analysis.
